# Effect of whey protein supplementation on levels of endocannabinoids and some of metabolic risk factors in obese women on a weight-loss diet: a study protocol for a randomized controlled trial

**DOI:** 10.1186/s12937-017-0294-x

**Published:** 2017-10-23

**Authors:** Fatemeh Haidari, Vahideh Aghamohammadi, Majid Mohammadshahi, Kambiz Ahmadi- Angali

**Affiliations:** 1 Department of Nutrition, Nutrition and Metabolic Diseases Research Center, Ahvaz Jundishapur University of Medical sciences, Ahvaz, Iran; 2 Department of Nutrition, Nutrition and Metabolic Diseases Research Center, Ahvaz Jundishapur University of Medical Sciences, Ahvaz, Iran; 3 Faculty of Public Health, Ahvaz Jundishapur University of Medical Sciences, Ahvaz, Iran

**Keywords:** Whey protein, Endocannabinoids, Weight loss, Obesity

## Abstract

**Background:**

Besides the effects of dietary long chain PUFA on circulating endocannabinoids concentrations, the impact of other nutrients on these system is not known and, whether changes in plasma endocannabinoids levels correlated with changes in body composition and biochemical metabolic risk factors in obese individuals, however, still remains to be characterized.

**Methods:**

We will conduct a 2 months’ open label, parallel-group, randomized controlled trial to determine the effect of whey protein supplementation on levels of endocannabinoids, glycemic and lipid profile, inflammatory factors, adipocytokines and body composition in 60 premenopausal obese women on a weight-loss diet.

**Conclusion:**

Due to strong relationship between endocannabinoids level and insulin resistance and obesity, in this trial, we will illustrate the other benefits of weight loss diet on health and metabolic risk factors. Also for the first, the effects of simultaneous weight loss diet and whey protein supplementation on these variables will be determined.

**Trial registration:**

Iranian Registry of Clinical Trials IRCT2017021410181N8.

**Electronic supplementary material:**

The online version of this article (10.1186/s12937-017-0294-x) contains supplementary material, which is available to authorized users.

## Background

Endocannabinoids (EC) identified to date are arachidonic acid metabolites, the most widely studied ones being arachidonoyl ethanolamide or anandamide and 2-arachidonoylglyceride (2-AG). These lipid-like substances are enzymatically generated from membrane phospholipid precursors and are thought to act as autocrine/paracrine mediators. Once released, they are rapidly metabolized, anandamide being degraded primarily by fatty acid amid hydrolase, whereas 2-AG is metabolized by monoglyceride lipase [1]. Endocannabinoids act as CB1 and/or CB2 receptor agonists. Classical CB1 and CB2 receptor transmission is coupled through inhibitory G-proteins (Gi/o) to inhibit adenylyl cyclase (i.e., cyclic AMP formation) and calcium channels and activate potassium channels and mitogen-activated protein kinase [1, 2].

In the CNS, endocannabinoids via activation of CB1 receptors increases food intake by modulating the activity of hypothalamic neurons and, subsequently, the release of orexigenic and anorexigenic neuropeptides. Furthermore, CB1 receptor signaling affects reward and reinforcement circuits in the mesolimbic system, leading to a preference for highly palatable food [3]. The CB1 receptor is also present in peripheral organs important in the control of metabolism and activates anabolic pathways, preferring energy storage [4]. In abdominal obesity, the endocannabinoid system (ECS) is generally up-regulated in both central and peripheral tissues, as indicated by high EC. This hyperactive ECS can contribute to further fat accumulation by enhancing food intake as well as by favoring lipogenesis. The endocannabinoid system plays an important role in the regulation of energy homeostasis, specifically of glucose and lipid homeostasis, and represents an important link between obesity and insulin resistance [3–7].

In several studies, obese individuals displayed higher serum levels of EC than lean individuals. Plasma endocannabinoids are higher in obese women compared with obese men and there are a strong association between high plasma EC levels and visceral obesity and metabolic parameters [7–10]. Besides the effects of dietary long chain PUFA on circulating EC concentrations, the impact of other nutrients on these system is not known [8, 9]. Whether changes in plasma EC levels correlated with changes in body composition and biochemical metabolic risk factors in obese individuals, however, still remains to be characterized [11, 12].

One strategy to help retention of muscle mass and increase fat mass loss during energy restriction includes the consumption of dietary protein intake greater than the RDA of 0.8 g/kg/day [13, 14]. Epidemiological studies indicate that the consumption of milk and dairy products is associated with a lower risk of metabolic disorders and cardiovascular diseases. In particular, whey protein seems to induce these effects because of bioactive compounds such as lactoferrin, immunoglobulins, glutamine and lactalbumin. In addition, it is an excellent source of branch chained amino acids. Although studies are conflicting, it has been suggested as an approach for the prevention and treatment of obesity and diabetes in both humans and animals [15]. Given the putative role of whey protein, plasma EC concentration association with visceral obesity and metabolic profile and, due to the effect of whey protein on EC concentration is not clear, we decided to evaluate the effect of whey protein supplementation on levels of endocannabinoids, glycemic and lipid profile, inflammatory factors, adipocytokines and body composition in obese women on a weight-loss diet.

## Methods

### Design

**Fig. 1:**
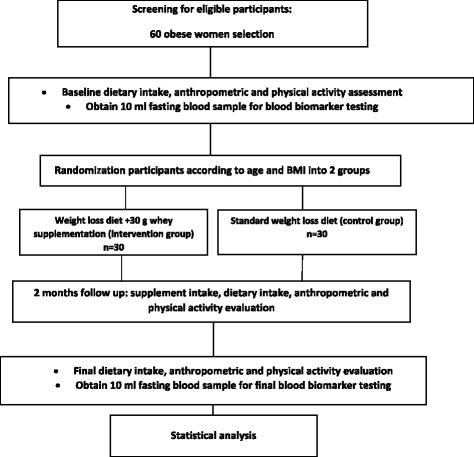
Protocol flow diagram; We will conduct a 2 months’ open label, parallel-group, randomized controlled trial to determine the effect of whey protein supplementation on levels of endocannabinoids, glycemic and lipid profile, inflammatory factors, adipocytokines and body composition in 60 premenopausal obese women on a weight-loss diet

**Fig. 2:**
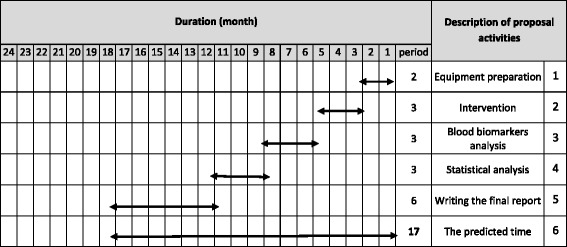
Timeline of study; we predicted 17 month for this trial

We will carry out a 2 months’ open label, parallel-group, randomized controlled trial. The proposed clinical trial will be held at the Nutritional research center, department of Nutrition, Ahvaz Jundishapur University of Medical Science for 2 months to assess the efficacy of daily whey 30 mg supplementation in obese subjects Figs. 1 and 2.

### Aims and study hypotheses

The primary aim of the current study is to examine the 2-month effects of whey protein supplementation on levels of endocannabinoids, glycemic and lipid profile, inflammatory factors, adipocytokines and body composition in obese women on a weight-loss diet. As a secondary aim, this study will also investigate the associations between changes in endocannabinoids concentrations and other variables. It is also expected that there will be an improvement in investigated variables.

### Participants

Subjects will be 60 premenopausal obese women aged 18 years or more. The following inclusion criteria will be applied: aged 18 years and older, BMI range of 30 to 40 kg/m^2^, absence of menopause, lactation, pregnancy and food allergies, not having cancer, hepatic, renal, thyroid and gastrointestinal disorders, no surgery for weight loss, no weight loss over the past 6 months, no taking herbs and drug that reduce appetite and weight and vitamin-mineral supplements. The participants with any of the following criteria will be excluded: become pregnant during study, unwilling to continue. At the end of the intervention, if the remained powders exceed 10% of total administered powders, that subject will be excluded from the study.

### Ethics and trial registration

The patients who meet the inclusion criteria will be fully informed about the study’s protocol. This protocol, approved by Medical Ethics Committee of Ahvaz University of Medical Sciences, is in accordance with the Declaration of Helsinki (approval number: IR.AJUMS.REC.1395.729). Each subject will sign an informed consent form. This investigation was registered on Iranian Registry of Clinical Trials (Irct registration number: IRCT2017021410181N8).

### Sample size

The sample size was calculated according to the changes in anandamide level, in response to weight loss, based on the study conducted by Mallipedhi et al. [16]. It was calculated considering 95% confidence interval, 80% power (α = 0.05 and β = 0.2), and the related mean and SD of anandamide levels in the mentioned study (μ1 = 0.297; μ2 = 0.209; SD1 = 0.042; SD2 = 0.049). Finally, 30 subjects were recruited for each group.

### Randomization

Eligible subjects will be stratified according to age and BMI randomly assigned to one of two groups: **control** (standard weight loss group, *n* = 30) and **intervention** (whey supplementation weight loss group, *n* = 30).

### Intervention

All participants will follow a hypocaloric diet of 800 kcal below estimated energy requirements, energy needs is estimated by Mifflin Jeor St equation. Control group will follow a hypocaloric diet of 800 kcal below estimated energy requirements and intervention group will follow a hypocaloric diet of 916 kcal below estimated energy requirements, each of intervention group subjects will receive 30-g whey protein powders daily. The powders will be provided in a sachet form (each sachet containing 30-g whey protein). Each 30 g of the whey protein supplement will be contained 116 kcal, 0.5 g of lipid, 0.4 g of carbohydrate, and 27.5 g of protein. Participants will be instructed to add one sachet to 250 ml cold water and consume immediately, every evening. In control and intervention group the macronutrients percent will be carbohydrate 55%, fat 30% and protein15%. Subjects will be asked to record powder intake and to check compliance, they will be followed by the dietitian via phone calls or SMS, every three days and, powders will be given to the subjects every 15 days. To create a variety in the diet while maintaining the general principles of diet, the same dietitian will give a dietary exchange list and a diet according to their food habits. A trained dietitian will design the energy requirements and macronutrient distribution and the same dietitian will be available to train participants on the diet.

### Measurements

A questionnaire about patients’ demographic situations, diseases, and medications, diabetes history and probable supplementations will be recorded at the beginning of the study. Dietary intake will be assessed by 3 days 24-h recall questionnaires (2 week days and 1 weekend day) at the baseline, middle and the end of study. Total energy and macronutrients intake will be calculated by using Nut IV (the Hearst Corporation, San Bruno, CA). To assess physical activity levels the International Physical Activity Questionnaire (IPAQ) will be used at the baseline and the end of study. Anthropometric parameters will be measured after overnight fasting with minimal clothing and without shoes. In all visits (every 15 days), body weight will be measured with the accuracy of 100 g using seca scale. Stature will be measured in a relaxed position using seca stadiometer with accuracy of 0.5 cm. BMI will be calculated as body weight (kg) divided by the square of height(m). Waist circumference will be assessed at above the iliac crest, just below the lowest rib margin at the end normal expiration to the nearest 0.5 cm. TANITA BC-418 body composition analyzer will be used to calculate total body fat and fat percent, fat free mass and fat free mass percent.

At baseline and end of the trial, 10 ml of venous blood samples (2 ml in EDTA- coated sterile tubes and 8 ml in regular tubes) will be collected after 10–12 h overnight fasting. For determination of plasma AEA and 2-AG, the blood in EDTA coated tubes will be centrifuged at 1500 g at 4 °C for 15 min and will be stored at -80 °C. These variables will be quantified by liquid chromatography/in-line mass spectrometry [7]. Regular tubes will be used for biochemical analyses such as determination of glucose, insulin, total cholesterol, HDL-C, triglyceride, adiponectin, leptin, TNF-α and IL-6 in serum. Blood glucose and lipid profile will be determined by enzymatic method with kits from Pars-azmoon (Tehran, Iran). LDL-C levels will be calculated by the Friedewald equation. Circulating insulin will be measured by immunoassay. Homeostasis model assessment – insulin resistance(HOMA-IR) will be calculated by the following formula: fasting glucose(mg/dl) × fasting insulin(μu/ml)/405. ELISA kits will be used to measure serum adiponectin and leptin levels.

### Analysis

SPSS version 17 (SPSS Inc., Chicago, IL, USA) will be used for data analysis. All data will be presented as mean ± SD. The percent change of each variable will be also calculated by the formula [(E − B)/B × 100], where E is the end of treatment values and B is the baseline values. The normality of variables will be tested using Kolmogorov- Smirnov test. Independent sample t-test and the paired sample t-test will be applied for comparing parametric continuous data between and within the groups, respectively. Mann-Whitney test and Wilcoxon test will be used to test the differences in asymmetric variables between and within the groups, respectively. To control confounding variables, analysis of covariance (ANCOVA) test were used to determine the differences between the two groups post-intervention, while adjusting for baseline measurements and covariates. A *p* value less than 0.05 will be considered to be statistically significant.

## Discussion

The discovery of the endocannabinoid system (ECS) and its influence on the regulation of energy homeostasis represents a significant advance in the study of obesity and type 2 diabetes. It seems circulating anandamide and 2-arachidonoyl-glycerol concentrations are correlated with increased of metabolic risk factors particularly in patients with abdominal adiposity. Weight loss interventions may modulate obesity-associated risk through interaction with the peripheral ECS. In daily life, successful loss of body weight by means of energy restriction will be reached by an energy-inefficient diet with high-satiety capacity. Regarding the macronutrient composition of the diet, elevated protein diets have gained interest. The effect of protein source during weight loss requires further study; thus, we will determine circulating endocannabinoid levels and other biochemical indices before and after 2 months of standard weight loss diet along with whey protein supplementation (Additional file 1: Table S1).

### Trial status

This trial is in the recruitment stage.

## Additional file

Additional file 1: Table S1. SPIRIT 2013 Checklist: Recommended items to address in a clinical trial protocol and related documents. (DOC 121 kb)

## Abbreviations

EC: endocannabinoids
ECS: endocannabinoid system
IPAQ: International Physical Activity Questionnaire
PUFA: polyunsaturated fatty acid
